# A twisted tale-radiological imaging features of COVID-19 on ^18^F-FDG PET/CT

**DOI:** 10.1186/s41824-020-00082-y

**Published:** 2020-07-22

**Authors:** Hazel O’Neill, Simon Doran, Francesco Fraioli, Afshin Nasoodi

**Affiliations:** 1 Department of Radiology, St. James’s Hospital, D08 X4RX Dublin, Ireland; 2 University College London Hospitals (UCLH), 5th Floor UCH, 235 Euston Rd, London, NW1 2BU UK

**Keywords:** COVID-19, ^18^F-FDG PET/CT

## Abstract

The COVID-19 pandemic has had a major impact on health care systems across the globe in a short period of time. There is a growing body of evidence surrounding the findings on hybrid imaging with FDG-PET/CT, and this case highlights the importance of molecular imaging in better understanding of the biomarkers of the disease which ultimately determine the success in building a model to predict the disease severity and monitoring the response to treatment.

## Introduction

December 2019 saw the first appearance of the 2019 novel coronavirus disease (COVID-19) caused by SARS-CoV-2 in China (Zhu et al. 2020). With the virus rapidly galloping across the globe, there has been an explosion of publications describing the imaging features of the infection with the bulk of published data focused on characterising ‘typical’ radiological manifestations of the disease on the respiratory system and potentially other organs (Zhou et al. 2020; Jajodia et al. 2020). The aim of this case report is to illustrate the radiological features of the infection and comment on patterns which may be used as imaging surrogates of disease activity and severity in vivo based on ^18^F-FDG PET/CT (FDG-PET/CT) findings in a critically ill patient infected with COVID-19 in support of growing literature available on this field (Qin et al. 2020; Zou and Zhu 2020; Kirienko et al. 2020).

## Case description

**Fig. 1:**
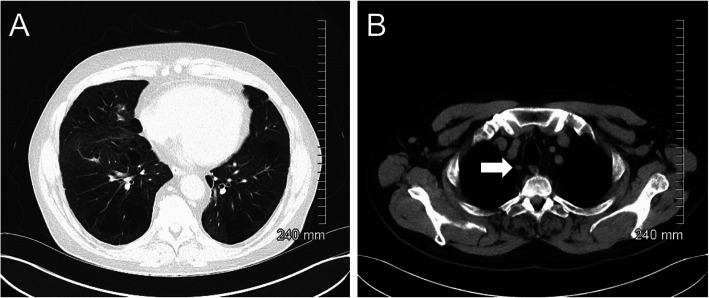
Initial CT thorax demonstrated no acute pulmonary infiltrates or lymphadenopathy (**a**). A morphologically normal ATS station 2R lymph node is highlighted by the white arrow (**b**)

**Fig. 2:**
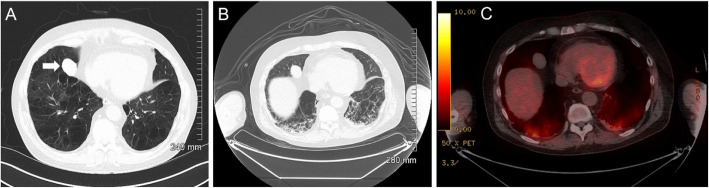
Initial CT thorax detected a right middle lobe mass (**a**). Subsequent PET-CT demonstrated no FDG uptake **b** (CT) and **c** (fused PET-CT)

A 63-year-old Caucasian male with a background of sarcoidosis and factor VIII deficiency was admitted to hospital in early March 2020 for workup ahead of commencing immunosuppressive treatment. CT thorax was performed to assess the known pulmonary sarcoidosis and identify any underlying infection ahead of potentially commencing immunosuppression. The CT at baseline demonstrated no acute pulmonary parenchymal abnormality or lymphadenopathy (Fig. 1); however, an incidental 3.4 cm right middle lobe mass was noted (Fig. 2; Image A) and a PET/CT was requested for further characterisation.

**Fig. 3:**
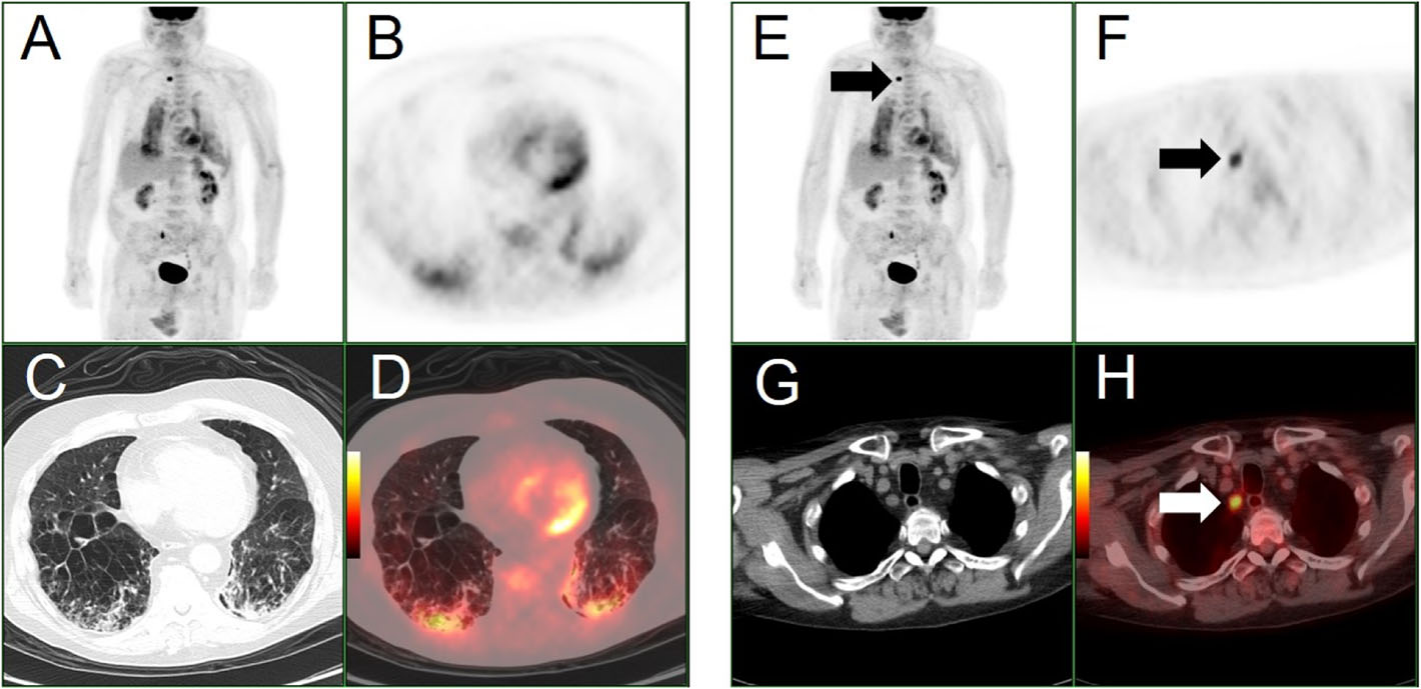
**a** PET maximum intensity projection (MIP), **b**–**d**: axial attenuation corrected PET, axial CT lung windows and axial fused PET/CT images through the lower thorax. **e** PET maximum intensity projection (MIP), **f**–**h**: axial attenuation corrected PET, axial CT mediastinal windows and axial fused PET/CT images through the superior mediastinum. These images highlight intense bilateral parenchymal metabolic activity, more prominently in the right lower lobe (images **a**–**d**) and high uptake in an ATS station 2R lymph node (**e**–**h**). Please note spurious right ureteric activity on the MIP images (images **a** and **e**)

In the run up to the PET/CT investigation, the patient became progressively unwell with the development of a febrile illness and increased oxygen requirement secondary to type I respiratory failure. PET/CT (GE Discovery VCT; USA), performed 9 days following the initial CT thorax, demonstrated no FDG uptake in the right middle lobe mass, likely a benign entity (Fig. 2b, c). There were however other unexpected findings which included intensely FDG positive, predominantly mid- to lower zone, diffuse bilateral subpleural intra- and interlobular septal thickening and ground-glass opacification, with patchy areas of a higher intensity of FDG uptake in the bases associated with consolidation (Fig. 3a–d). More interestingly, intense FDG uptake was present within an American Thoracic Society (ATS) station level 2R mediastinal lymph node with SUVmax of 8.7 (Fig. 3e–h). There was no other FDG-positive abnormality outside the thorax.

Due to his increased vulnerability to opportunistic infection secondary to immunosuppression, and current pandemic of COVID-19 and unbeknown to the PET/CT Department staff, the patient had undergone laboratory testing (Altona RealStar® SARS-CoV-2 RT-PCR Kit 1.0) for the virus in the time interval between the requesting and performance of PET/CT and subsequently received a positive diagnosis for COVID-19. Unfortunately, the patient’s clinical condition deteriorated and he passed away 1 week following PET/CT. One senior member of staff in the PET department, who had come in close contact with the patient required self-isolation for 2 weeks. Consequently, all staff received training to deal with potential COVID-19 patients including hygiene and disinfection procedures, handling and moving patients, use of personal protective equipment and restricted contacts in order to maximally reduce the risk of transmission.

## Discussion

In our case, COVID-19 was suspected at the time of interpretation of the PET/CT images, without the knowledge of a positive RT-PCR test, but rather based on the rapid development of extensive pulmonary infiltrates in a relatively short interval since the unremarkable baseline CT, and the patient’s risk profile. The findings were significant but certainly unexpected given the clinical question of characterisation of a potential lung malignancy, which in this case was proven to be a red herring and possibly secondary to an intrapulmonary haematoma related to bleeding diathesis. A number of key issues emerge from this case.

Firstly, assessment for COVID-19 was not the primary objective and resulted in unnecessary exposure of the staff in the PET/CT department to the virus during the transfer and preparation of the patient, which ideally should have been postponed pending a negative test result. This case highlights the potential role of nuclear medicine departments in the current pandemic in the detection of unexpected COVID-19 in patients scanned for other indications. Importantly, as for our case, the majority of PET/CT studies are performed for oncology patients, often on immunosuppressive therapy, and therefore requiring urgent medical attention and contact tracing within oncology units (Albano et al. 2020). It also reinforces the importance of close patient movement monitoring throughout the hospital during the time interval between testing suspected cases and laboratory results (Albano et al. 2020; Lu et al. 2020).

Next, the imaging findings on the CT component of the study were in line with the other case reports available in the literature and included a crazy-paving pattern with areas of ground-glass infiltration and consolidation in a subpleural distribution (Zu et al. 2020). Furthermore, the extent and intensity of FDG uptake in the lungs, concordant with the areas of CT abnormality, were thought to be indicative of active COVID-19 infection (Zu et al. 2020). Having since imaged a number of other confirmed or suspected cases with less pronounced FDG activity on PET/CT, and particularly since “casual FDG uptake” has been shown to be a feature of COVID-19 infection in a small case series of asymptomatic patients in a recent publication, the possibility of a more aggressive strain of the virus, and patient’s immunosuppression may have been the contributing factors (Setti et al. 2020). Based on our findings and limited available evidence from other investigators, there may be a role in the future for PET/CT in the management of patients during the endemic phase to assist in the differential diagnosis, differentiating active from non-active disease and assessment of the response to treatment (Deng et al. 2020). Critically, the location of the intensely FDG-positive normal size upper mediastinal lymph node, demonstrated in our case study, was discordant with the distribution of the pulmonary parenchymal involvement. This may suggest that lymphadenopathy could be an independent imaging biomarker of the disease, similar to what demonstrated for other infections (Chadburn et al. 1989). Indeed, based on limited data, lymphadenopathy has been suggested as a prognostic marker of COVID-19 infection severity (Valette et al. 2020) and a predictor of a worse outcome (Sardanelli et al. 2020). In view of the grave clinical outcome of our patient, there may be an argument in support of a further prognostic role of higher SUVmax values of FDG positive nodal disease, as a biomarker which could herald a more severe case of the infection with the virus.

Finally, the presence of nodal uptake, in a more suggestive setting of malignancy such as lymphoma or lung tumour, could have erroneously resulted in over-staging of the nodal disease (Zanoni et al. n.d.).

## Conclusion

CT is the primary cross-sectional diagnostic imaging modality of choice in the assessment of severe cases when clinically indicated. This case study however highlights the importance of the metabolic activity provoked in the lung parenchyma by COVID-19 infection which could be illustrated only with PET/CT. It also underlines the potential value of PET/CT and molecular imaging in the future, in determining disease activity and severity based on some of the recent observations and limited available data. However, until a clear role for PET/CT in COVID-19 is established through further research, unnecessary potential exposures to staff and other patients should be avoided by careful screening of the patients for COVID-19 infection in suspected cases prior to PET/CT for malignancy workup, particularly when the primary is in the thorax to avoid pitfalls.

## Data Availability

Not applicable.
